# Acute juvenile Paracoccidioidomycosis: A 9-year cohort study in the endemic area of Rio de Janeiro, Brazil

**DOI:** 10.1371/journal.pntd.0005500

**Published:** 2017-03-29

**Authors:** Priscila Marques de Macedo, Rodrigo Almeida-Paes, Dayvison Francis Saraiva Freitas, Andréa Gina Varon, Ariane Gomes Paixão, Anselmo Rocha Romão, Ziadir Francisco Coutinho, Claudia Vera Pizzini, Rosely Maria Zancopé-Oliveira, Antonio Carlos Francesconi do Valle

**Affiliations:** 1 Infectious Dermatology Clinical Research Laboratory, Evandro Chagas National Institute of Infectious Diseases, Fiocruz, Rio de Janeiro, Brazil; 2 Mycology Laboratory, Evandro Chagas National Institute of Infectious Diseases, Fiocruz, Rio de Janeiro, Brazil; 3 Department of Inpatient Health Care, Evandro Chagas National Institute of Infectious Diseases, Fiocruz, Rio de Janeiro, Brazil; 4 Geoprocessing Laboratory, Institute of Scientific and Technological Communication and Information in Health, Fiocruz, Rio de Janeiro, Brazil; 5 Sergio Arouca National School of Public Health, Fiocruz, Rio de Janeiro, Brazil; University of California San Diego School of Medicine, UNITED STATES

## Abstract

**Background:**

Paracoccidioidomycosis (PCM) is a systemic mycosis caused by pathogenic dimorphic fungi of the genus *Paracoccidioides*. It is the most important systemic mycosis in Latin America and the leading cause of hospitalizations and death among them in Brazil. Acute PCM is less frequent but relevant because vulnerable young patients are affected and the severity is usually higher than that of the chronic type.

**Methods:**

The authors performed a retrospective cohort study from 2001 to 2009 including acute juvenile PCM patients from a reference center in Rio de Janeiro, Brazil. Clinical, epidemiological, diagnostic, therapeutic, and prognostic data were reported.

**Results:**

Twenty-nine patients were included. The average age was 23 years old and the male to female ratio was 1:1.07. All cases were referred from 3 of 9 existing health areas in the state of Rio de Janeiro, predominantly from urban areas (96.5%). Lymph nodes were the most affected organs (100%), followed by the skin and the spleen (31% each). Twenty-eight patients completed treatment (median 25 months) and progressed to clinical and serological cure; 1 death occurred. Twenty-four patients completed 48-month median follow-up. Four patients abandoned follow-up after the end of treatment. The most frequent sequela was low adrenal reserve. *Paracoccidioides brasiliensis* S1 was identified by partial sequencing of the *arf* and *gp43* genes from 4 patients who presented a viable fungal culture.

**Conclusion:**

Acute juvenile PCM is a severe disease with a high rate of complications. There are few cohort clinical studies of acute PCM in the literature. More studies should be developed to promote improvement in patients’ healthcare.

## Author summary

Paracoccidioidomycosis (PCM) is a neglected systemic mycosis caused by fungi of the genus *Paracoccidioides* present in the soil and is endemic to Latin America. The acute clinical form of this disease is less frequent than the chronic type of presentation. However, it is a more severe clinical condition, potentially life-threatening, affecting many important organs of the immune defense such as the lymph nodes, liver, and spleen. It can lead to serious complications as well as many sequelae in young vulnerable patients and children. There are few works published in the literature concerning clinical, epidemiological, prognostic and therapeutic features of acute juvenile PCM. This study aims to describe a 9-year cohort study of patients with acute juvenile PCM from a reference center in the endemic area of Rio de Janeiro, Brazil. Results demonstrate that early diagnosis can prevent poor outcomes and that a specialized medical structure is required to promote proper healthcare for these patients. Severe outcomes such as low adrenal reserve and a death occurred in 4 and 1 patients, respectively. The authors expect that these results can contribute to a better understanding of this severe fungal disease.

## Introduction

Paracoccidioidomycosis (PCM) is a severe systemic mycosis endemic to Latin America [1]. In Brazil, it is the leading cause of hospitalizations and death among all systemic mycoses in immunocompetent patients and an important cause of morbidity [2–4]. Primary pathogenic dimorphic fungi of the genus *Paracoccidioides* are the etiological agents of this disease and, according to the literature; the remarkable genetic diversity between phylogenetic species seems to cause variations in clinical presentation, therapeutic response, diagnosis, and prognosis [5–7]. These hypotheses are based on observations of a few case reports with molecular identification of the fungus [8–11]. Acute PCM, also known as juvenile-type PCM, corresponds to 3% to 5% of all PCM cases but is considered the most severe clinical form of this mycosis because it affects vulnerable young or, less frequently, immunocompromised people, usually presenting as a disseminated disease involving the mononuclear phagocyte system including the lymph nodes, liver, spleen, and bone marrow [12]. It is frequently characterized by significant consumptive syndrome and massive cervical adenopathy that can be initially misdiagnosed as lymphoma or tuberculosis. Acute PCM usually develops with some complications and sequelae. The present study aims to perform a descriptive analysis of epidemiological, clinical, therapeutic, and prognostic data in a cohort of patients with acute PCM, evaluated in a reference center for this mycosis in Rio de Janeiro state, Brazil, an important endemic area. There are few detailed cohort clinical studies concerning this type of clinical form in the literature because of its low incidence and due to its environmental, geographical, and occupational exposure variability [13–15]. Furthermore, molecular identification of available *Paracoccidioides* strains is provided to contribute to a better understanding of this challenging subject.

## Methods

### Ethical statements

Evandro Chagas National Institute of Infectious Diseases Research Ethics Committee has approved this study protocol under the register CAAE 42590515.0.0000.5262. The patients’ data were anonymized/de-identified to protect patients’ privacy/confidentiality.

### Study design and area

**Fig 1:**
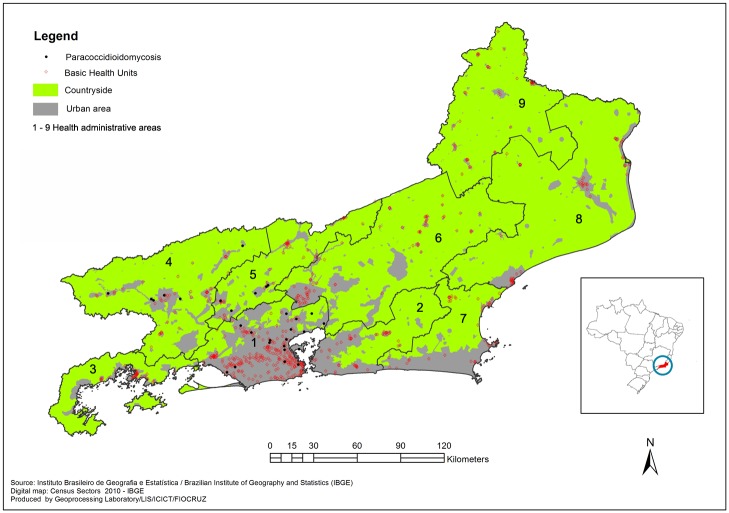
Map of the state of Rio de Janeiro showing georeferenced PCM cases from this study according to health access and urban-rural distribution.

This is a retrospective cohort study from a reference center for PCM clinical assistance in the state of Rio de Janeiro, an important endemic area for this mycosis in Brazil. Rio de Janeiro is a Brazilian state located in the southeast of the country, with the highest demographic density in Brazil. Rio de Janeiro is divided into 92 municipalities; 5 are predominantly rural, and 81 have habitants living in rural zones [16]. In 2013, the state was divided into 9 regions known as health areas based on demographic and socio-economic data to better understand and to plan strategic health policies (Fig 1).

### Patients

All cases of acute PCM admitted to INI/Fiocruz from 2001 to 2009 were included in this study. Inclusion criteria were diagnosis of PCM by direct examination, culture or histopathology, and classification of the acute form by clinical findings based on a consensus in PCM [12]. Medical records of these patients were collected, and information concerning epidemiological, clinical, and therapeutic data was documented on a standardized anonymized clinical report form.

### Diagnosis

Direct examination of clinical samples was performed with KOH 10%. Cultures were performed on modified Sabouraud dextrose agar and mycobiotic agar at 25°C. Suspected *Paracoccidioides* cultures were subcultured in Fava-Netto agar at 37°C for dimorphism confirmation. Serology for PCM was performed using Ouchterlony double immunodiffusion (ID).

Histopathological examination of tissues was performed using hematoxylin-eosin (H&E) and other staining techniques such as Grocott's methenamine silver stain (GMS) or Periodic acid—Schiff (PAS) for a better visualization of fungal parasitic structures.

All patients were given a standard routine clinical evaluation including physical examination, blood tests (hematology and biochemistry), parasitological stool analysis, bacterial microbiologic analysis of sputum (3 samples for acid-fast bacilli and culture), chest radiograph, and other imaging exams when indicated (brain computerized tomography [CT], and abdominal CT or ultrasonography).

Adrenal function was evaluated using the ACTH (Cortrosyn) stimulation test. Low adrenal reserve was defined as a normal basal level without reaching at least 20 mg/dl after 30 and 60 minutes of stimulation. Glandular insufficiency was defined as reduced basal levels associated with clinical symptoms (extreme fatigue, skin hyperpigmentation, low blood pressure, fainting, hypoglycemia, nausea, diarrhea or vomiting, and abdominal pain).

### Therapeutic regimen

The therapeutic regimen was based on consensus in PCM [12]. Sulfamethoxazole/trimethoprim (SMZ-TMP), itraconazole and amphotericin B were the standard drug therapy. Combination of drugs was administered in cases of refractory, poor outcome and severe clinical conditions such as neurological complications.

### Prognostic data

The grade of severity was based on a standard classification proposed by Mendes [17]. Cure criteria were clinical, serological and radiological according to those defined in the consensus as well as the periodicity of clinical and laboratorial evaluations [12]. The recommended time of follow-up was 24 to 60 months after the end of treatment.

### Molecular identification

Genomic DNA was extracted from the yeast phase of viable *Paracoccidioides* cultures obtained at the time of the patients’ diagnosis. Amplicon products of polymerase chain reaction (PCR) using 2 protein-encoding genes *arf* (ADP ribosylation factor) and *gp43* (glucan 1,3-beta-glucosidase) primers were submitted to automated partial nucleotide sequencing in the Platform PDTIS/FIOCRUZ [10]. A BLAST (Basic Local Alignment Search Tool) analysis (www.ncbi.nlm.nih.gov/BLAST) was performed comparing these sequences to those from isolates belonging to the *Paracoccidioides brasiliensis* complex previously deposited by Matute et al.

### Statistics

Statistical analysis was conducted using Stata 12. The data were summarized as percentages for categorical variables and median for continuous variables.

## Results

Twenty-nine patients fulfilled the inclusion criteria. Clinical, demographic, and prognostic data of these patients are summarized in Table 1. Therapeutic and serological data are presented in Table 2.

**Table 1:** Demographic, clinical, and prognostic profile in 29 acute juvenile PCM patients.

	n	%
**Sex masculine/feminine**	14/15	48.3/51.7
**Race (skin color)**		
White	10	34.5
Mixed	10	34.5
Black	9	31.0
**Grade of severity of the disease**		
Moderate	13	44.8
Severe	16	55.2
**Coinfections**		
Intestinal worms	5	17.2
Tuberculosis	3	10.3
HIV	1	3.4
HCV	1	3.4
**Complications**		
Anemia	24	82.7
Hypoalbuminemia	16	55.2
Low adrenal reserve	5*	17.2
Cholestasis	3	10.3
Colon stenosis	1	3.4
**Sequelae**		
Low adrenal reserve	4	13.8
Lymphedema	2	6.9
Spleen calcifications	1	3.4
Keloids	1	3.4
**Mortality**	1	3.4

*Two patients with low adrenal reserves were among 4 patients with molecular identification of the strain (*Paracoccidioides brasiliensis* S1) KX463649, KX463650 and KX463653, KX463654. Recovery of the adrenal function occurred in 1 patient. The other 2 patients had no sequelae.

**Table 2:** Serological and therapeutic results in 29 acute juvenile PCM patients.

	Median	Min-Max
Serology titers (1:)*		
Before treatment	8	0**–256
End of treatment	0	0–8***
**Time of treatment (months)**	25	6–75
Time of follow-up (months)****	48	12–108

*One patient without available serology titers.

**Five patients with negative results before, during, and after treatment.

***Two patients with positive results after the end of the treatment: 1 abandoned and the other had a serology scar (1:2).

****Four patients abandoned after the end of treatment.

The average age was 23 years (minimum 8, maximum 44). Students and general services assistants were the 2 occupational labors reported. Rural activity was reported in 1 case. All 29 cases from this study were referred from 3 of 9 health areas in the state of Rio de Janeiro, predominantly from the urban area (Fig 1). All these patients were born in Rio de Janeiro state except 2 patients who were from Bahia and Minas Gerais states (northeast and southeast, 1640 and 500 km from Rio de Janeiro, respectively). There was no report of travels to other regions before the symptoms began. The median time of symptoms’ onset until PCM diagnosis was 4 months (minimum 2, maximum 84). Diagnostic confirmation of PCM occurred most frequently (72.4%) through clinical specimen analysis obtained by invasive methods such as biopsies (Table 3).

**Table 3:** Clinical specimens from which PCM was diagnosed.

Clinical specimen	n	%
Lymph node biopsy (histopathology and mycological analyses)	17*	44.8
Lymph node aspirate (mycological analysis)	3	10.3
Skin biopsy (histopathology and mycological analyses)	3**	10.3
Oral mucosa shaving/biopsy (mycological analysis)	3	10.3
Sputum (mycological analysis)	2***	6.9
Intestinal biopsy (histopathology analysis)	1	3.4
Serum (ID serology test)	1****	3.4

*Fourteen samples by histopathology, 2 by mycological analysis, and 1 by both techniques.

**Two samples by histopathology and 1 by mycological analysis.

***One patient also presented a positive mycological analysis from a lymph node biopsy.

****This patient presented 2 negative sputum samples by mycological analysis.

**Fig 2:**
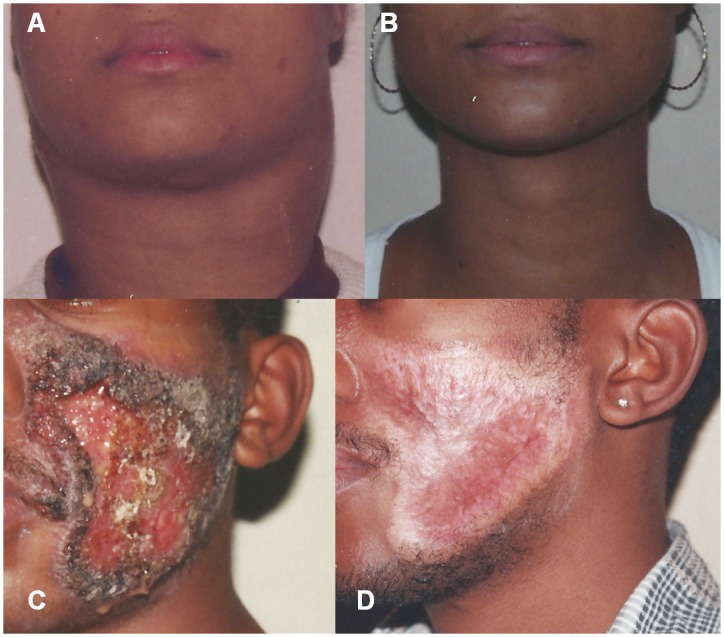
Lymph node and skin involvement in 2 patients from the present study. (A) Cervical lymph node enlargement with a “bull’s neck” appearance in a female patient. (B) The same patient after 35 months of amphotericin B, itraconazole, and sulfamethoxazole/trimethoprim treatment. (C) Extensive ulcerative skin lesions on the face of a male patient. (D) The same patient after 48 months of sulfamethoxazole/trimethoprim treatment. Photographs by ACFV were obtained for registration of the patients’ recovery. Both individuals agreed to have their photographs taken and published.

The lymph nodes were the most affected organs (100%), followed by the skin (31%), the spleen (31%), and the liver (27.6%). Fig 2 shows 2 patients from this study presenting lymph nodes enlargement and skin lesions, before and after treatment. The adrenals and the central nervous system (CNS) were affected in 5 and 2 patients, respectively. The frequency of other organs involvement is detailed in Table 4.

**Table 4:** Affected organs in acute juvenile PCM cases from this study.

Organ	n	%
Lymph nodes	29	100,0
Skin	9	31.0
Spleen	9	31.0
Liver	8	27.6
Adrenals	5	17.2
Oral Mucous	4	13.8
Lungs	3*	10.3
CNS	2	6.9
Pancreas	2	6.9
Large intestine	1	3.4

*Two with mycological confirmation by fungal isolation from the sputum and the other probable since other causes such as pulmonary tuberculosis were ruled out.

**Fig 3:**
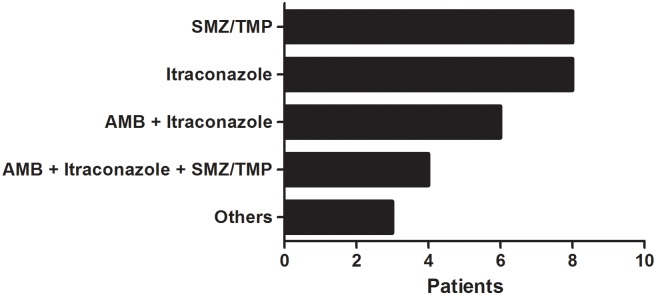
Therapeutic regimen prescribed for the treatment of 29 acute juvenile PCM cases from this study. AMB (amphotericin B) and SMZ/TMP (sulfamethoxazole/trimethoprim). Others: itraconazole + SMZ/TMP; AMB + SMZ/TMP; and AMB + fluconazole + SMZ/TMP.

The most common clinical complication was low adrenal reserve, while the most frequent laboratory abnormalities observed were anemia and hypoalbuminemia. Colon stenosis leading to intestinal obstruction occurred in 1 patient. Low adrenal reserve was the most frequent sequela requiring indefinite steroid replacement therapy. Hospitalization was necessary for 20 patients to promote intensive healthcare support and/or intravenous therapy with amphotericin B. Coinfections were detected in 8 patients: 3 cases of pulmonary tuberculosis (TB) from which 1 also presented hepatitis C (HCV), and 1 case of human immunodeficiency virus (HIV) infection. The other patients presented intestinal worm infections. Twenty-eight patients completed treatment (median 25 months) and progressed to clinical and serological cure; 1 death due to neurological PCM occurred. Itraconazole and SMZ-TMP in monotherapy prevailed followed by different types of drug associations (Fig 3).

Twenty-four patients completed follow-up after the treatment and 4 abandoned it. *Paracoccidioides brasiliensis* S1 was identified by the partial sequencing of the *arf* and *gp43* gene regions from 4 patients from whom we could retrieve a viable culture. Two of these 4 patients presented a moderate clinical condition without complications or sequelae, while the other 2 cases were considered severe since the adrenals were affected, although the recovery of adrenal’s function occurred in 1 patient.

## Discussion

Acute juvenile PCM is a severe presentation of this neglected systemic mycosis that most frequently affects vulnerable young patients with low socioeconomic status and can lead to life-threatening clinical conditions, serious complications, hospitalization, and permanent sequelae. The infection is acquired via the inhalation of *Paracoccidioides* filamentous propagules present in the soil of endemic areas but also probably dispersed by the wind and influenced by other climatic features [18]. Thus, infection easily occurs in the countryside and is usually related to rural activities, although susceptible individuals can be found in the urban areas without this occupational profile as observed in classical acute PCM and confirmed by sociodemographic data of the present study. These patients present a specific cellular immunodeficiency against the fungal agent and the inability to develop a granuloma response leads to acute/subacute infection’s progression soon after hematogenous spread. The mononuclear phagocytic system is the main site of infection. In the literature, the frequency of organs involved is as follows: the lymph nodes, the digestive tract, the liver, the spleen, bones, joints and the skin [12,19]. In this study, the lymph nodes were affected in all cases, followed by skin lesions and hepatic/spleen involvement. Acute PCM cases without lymph nodes involvement are rare and challenging to diagnose [10]. Cases with bone and joints commitment were not observed, but important organs such as the adrenals and the CNS were affected in the patients from the casuistic studied. Lymph abdominal presentation was once thought to be related to *Paracoccidioides lutzii* infection [6], although this is based on clinical sporadic observation and a lack of studies with consistent statistical data does not allow inferences about species-specific clinical manifestations. In this work, although a few viable fungal cultures could be retrieved to allow a genetics evaluation, the results suggest that *P*. *lutzii* alone is not responsible for lymph abdominal, critical, and severe conditions, and perhaps the severity of the disease could be better explained by host-parasitic interaction [20,21], as proposed before in a published clinical and molecular severe case report in which the etiological agent involved was *P*. *brasiliensis* S1 [10]. The important immune profile in PCM physiopathology is applicable in this context. Acute and subacute PCM are characterized by high titers of secondary antibodies produced by lymphocytes B activation due to exacerbated Th2 cytokines responses such as IL4, IL5, and IL10 [22,23]. Thus, serology tests usually present high positivity in acute and severe cases [17]. The absence of antibody detection in Ouchterlony immunodiffusion tests can be explained by low titer production or differences in antigenic profiles obtained from distinct species to perform the test [24–28]. In this study, 3 strains identified as *P*. *brasiliensis* S1 were isolated from patients with positive serology tests. The other patient, whose strain was also identified as *P*. *brasiliensis* S1, presented negative results before, during, and after treatment despite the severity of the case. The small number of strains retrieved for molecular analysis is a limitation of this study although these data reveal that the immune response in PCM needs to be further explored. Epidemiological factors and immune response patterns can justify the presence of intestinal worm coinfection. The incidence of TB and PCM coinfection reported in the literature is about 5% to 19% [29,30], similar to the data detected in this study. However, pulmonary TB is mostly related to the chronic type of PCM. HIV and HCV infections are barely reported to coexist with PCM perhaps because of epidemiology aspects, since PCM is essentially a rural mycosis. The important superposition of other severe and endemic infections such as TB, HIV and HCV also highlights the vulnerability of these young patients.

Regarding prognostic data, the results presented here show high rates of hospitalization (almost 70% of cases) and the occurrence of a fatal outcome in 1 case. Therefore, the occurrence of severe sequelae such as low adrenal reserve requiring indefinite steroid replacement therapy is a worrisome problem [31]. Early diagnosis and treatment can prevent complications and poor outcomes. In the literature, the time from symptoms’ onset until health assistance access is about 1 to 3 months, while this study shows a 4-month period until diagnosis confirmation [12]. PCM diagnosis requires a specialized health multidisciplinary team and laboratory infrastructure. Invasive techniques are also required to obtain clinical samples for diagnostic confirmation. Almost 50% of the patients from this study were referred from heath areas with low access to specialized medical assistance (Fig 1). Complications related to diagnostic delay can be severe and even fatal, such as adrenal insufficiency, acute abdomen due to intestinal obstruction, seizures secondary to fungal brain tumors, and respiratory impairment [31–35]. These complications require a high-complexity multidisciplinary health assistance, including surgery and intensive care support. In this study, a satisfactory plan of treatment and follow-up accomplishments were obtained, since consensus recommends 6–24 months and 24 months, respectively [12]. This certainly contributed to reducing PCM morbimortality in the casuistic included in this study. Drug association is a good strategy for critical and neurological cases [36,37]. The authors encourage clinical research and more reports concerning acute PCM clinical experience to promote greater knowledge and assistance for this challenging, severe, and neglected infectious fungal disease.

All sequences generated in this study were deposited in GenBank^®^ (accession numbers KX463647, KX463648, KX463649, KX463650, KX463651, KX463652, KX463653, and KX463654).

## Supporting information

S1 Checklist STROBE checklist. (DOC)
