# Supplementary material for: Acute juvenile Paracoccidioidomycosis: A 9-year cohort study in the endemic area of Rio de Janeiro, Brazil
Source: PLoS Negl Trop Dis. 2017 Mar 29;11(3):e0005500. doi: 10.1371/journal.pntd.0005500 (PMC5386294; doi:10.1371/journal.pntd.0005500)
Supplement: S1 Checklist — (DOC) [file pntd.0005500.s001.doc]

STROBE Statement—Checklist of items that should be included in reports of ***cohort studies***

|  | Item No | Recommendation |
| --- | --- | --- |
| **Title and abstract** | 1 | (*a*) Indicate the study’s design with a commonly used term in the title or the abstract  Lines 1 and 2: Acute Juvenile Paracoccidioidomycosis: A 9-year **cohort study** in the endemic area of Rio de Janeiro, Brazil. |
|  |  | (*b*) Provide in the abstract an informative and balanced summary of what was done and what was found – Lines 33 to 57: The abstract includes background information, methodology description, summarized results data and a final conclusion. |
| Introduction | | |
| Background/rationale | 2 | Explain the scientific background and rationale for the investigation being reported  Lines 78 to 95 – provide current scientific information concerning the subject of the study including some hypotheses related to the theme. |
| Objectives | 3 | State specific objectives, including any prespecified hypotheses  Lines 95 to 103 – main and secondary objectives are reported in this topic. |
| Methods | | |
| Study design | 4 | Present key elements of study design early in the paper  Lines 110 to 112 – describe details of the study design |
| Setting | 5 | Describe the setting, locations, and relevant dates, including periods of recruitment, exposure, follow-up, and data collection  Lines 112 to 129 – the area and period of the present study as well as information concerning data collection are specified in this section. |
| Participants | 6 | (*a*) Give the eligibility criteria, and the sources and methods of selection of participants. Describe methods of follow-up.  Lines 124 to 126 – contain the inclusion criteria and also the selection tools referred.  Lines 159 to 162 – standardize and describe periodicity and time of clinical and laboratorial follow-up as well as cure criteria. |
|  |  | (*b*) For matched studies, give matching criteria and number of exposed and unexposed – Not applicable. |
| Variables | 7 | Clearly define all outcomes, exposures, predictors, potential confounders, and effect modifiers. Give diagnostic criteria, if applicable  Lines 130 -159 – Diagnostic, therapeutic and outcome description is provided. |
| Data sources/ measurement | 8* | For each variable of interest, give sources of data and details of methods of assessment (measurement). Describe comparability of assessment methods if there is more than one group.  Only one group is applicable. Variables of interest (epidemiological, clinical, prognostic and laboratory) were obtained from medical records and the method of measurement is detailed. Lines 126-150 |
| Bias | 9 | Describe any efforts to address potential sources of bias.  It is a descriptive study including all patients that fulfilled the inclusion criteria of the selected period and whose evaluation was standardized based on an international consensus. |
| Study size | 10 | Explain how the study size was arrived at. Not applicable. |
| Quantitative variables | 11 | Explain how quantitative variables were handled in the analyses. If applicable, describe which groupings were chosen and why. Not applicable. |
| Statistical methods | 12 | (*a*) Describe all statistical methods, including those used to control for confounding |
|  |  | (*b*) Describe any methods used to examine subgroups and interactions |
|  |  | (*c*) Explain how missing data were addressed |
|  |  | (*d*) If applicable, explain how loss to follow-up was addressed |
|  |  | (*e*) Describe any sensitivity analyses  * Descriptive study. Measures of frequency were obtained using Stata 12.  Loss of follow-up, when occurred, is explained in the manuscript. |
| Results | | |
| Participants | 13* | (a) Report numbers of individuals at each stage of study—eg numbers potentially eligible, examined for eligibility, confirmed eligible, included in the study, completing follow-up, and analysed  Line 177 – report the number of all patients diagnosed (gold standard – inclusion criteria).  Loss of follow-up and other outcomes details are specified in lines 238 to 264. |
|  |  | (b) Give reasons for non-participation at each stage. Not applicable. |
|  |  | (c) Consider use of a flow diagram. Not applicable. |
| Descriptive data | 14* | (a) Give characteristics of study participants (eg demographic, clinical, social) and information on exposures and potential confounders  Table 1 (line 180) summarizes demographic, social and clinical information; other data not applicable to the table were mentioned in the text (lines 196 to 205). |
|  |  | (b) Indicate number of participants with missing data for each variable of interest  When applicable, it was reported and detailed (line 187). |
|  |  | (c) Summarise follow-up time (eg, average and total amount)  These data are summarized in Table 2 (line 186). |
| Outcome data | 15* | Report numbers of outcome events or summary measures over time  These data are summarized in Table 1 (line 180). |
| Main results | 16 | (*a*) Give unadjusted estimates and, if applicable, confounder-adjusted estimates and their precision (eg, 95% confidence interval). Make clear which confounders were adjusted for and why they were included.  Not applicable. |
|  |  | (*b*) Report category boundaries when continuous variables were categorized  Not applicable. |
|  |  | (*c*) If relevant, consider translating estimates of relative risk into absolute risk for a meaningful time period.  Not applicable. |
| Other analyses | 17 | Report other analyses done—eg analyses of subgroups and interactions, and sensitivity analyses.  Not applicable. |
| Discussion | | |
| Key results | 18 | Summarise key results with reference to study objectives  Lines 273 to 277 – Results concerning epidemiological data  Lines 282 to 288 – Results concerning clinical data  Lines 288 – 297 – Results concerning molecular data  Lines 297 – 309 – Results concerning serological data |
| Limitations | 19 | Discuss limitations of the study, taking into account sources of potential bias or imprecision. Discuss both direction and magnitude of any potential bias.  Lines 309 to 311 – Limitation not related to bias since it was only described as a complementary data (molecular results). |
| Interpretation | 20 | Give a cautious overall interpretation of results considering objectives, limitations, multiplicities of analyses, results from similar studies, and other relevant evidence.  Lines 324 to 343 – Compiled results data were interpreted and compared with current scientific information published to conclude and suggest improvements in the subject as well as in patients’ healthcare. |
| Generalisability | 21 | Discuss the generalisability (external validity) of the study results.  Not viable from this moment. Specific scientific knowledge yet in construction. More studies are needed to better understanding of this severe, endemic and neglected disease (suggested in lines 341 to 343). |
| Other information | | |
| Funding | 22 | Give the source of funding and the role of the funders for the present study and, if applicable, for the original study on which the present article is based.  Financial support details were informed in online submission as instructions provided by the journal’s team. Funding was applied for laboratory reagents obtainment. The funders had no role in study design, data collection and analysis, decision to publish, or preparation of the manuscript. |

*Give information separately for exposed and unexposed groups.

**Note:** An Explanation and Elaboration article discusses each checklist item and gives methodological background and published examples of transparent reporting. The STROBE checklist is best used in conjunction with this article (freely available on the Web sites of PLoS Medicine at http://www.plosmedicine.org/, Annals of Internal Medicine at http://www.annals.org/, and Epidemiology at http://www.epidem.com/). Information on the STROBE Initiative is available at http://www.strobe-statement.org.
